# Mapping genomic regions for reproductive stage drought tolerance in rice from exotic landrace-derived population

**DOI:** 10.3389/fpls.2024.1495241

**Published:** 2025-01-07

**Authors:** Challa Venkateshwarlu, Paresh Chandra Kole, Arun Kumar Singh, Pronob J. Paul, Pallavi Sinha, Vikas Kumar Singh, Arvind Kumar

**Affiliations:** ^1^ South Asia Hub, International Rice Research Institute (IRRI), International Crops Institute for the Semi-Arid Tropics (ICRISAT) Campus, Hyderabad, Telangana, India; ^2^ Institute of Agriculture, Visva Bharati University, Bolpur, West Bengal, India

**Keywords:** reproductive stage drought, QTL mapping, linkage, grain yield, rice

## Abstract

In the rapid climate change scenario and subsequent rainfall patterns, drought has emerged as a bottleneck for crop production across crops, especially in rainfed rice. Drought significantly affects the development and production of most modern rice cultivars. Thus, recent breeding efforts have aimed to integrate drought tolerance traits in existing rice varieties through conventional and molecular approaches. The identification of grain yields quantitative trait loci (QTLs) under drought conditions, an important trait with high selection efficiency, may lead to the development of drought-tolerant rice varieties. The study reported the grain yield QTLs identified under the reproductive stage of drought stress in the F_2_-derived mapping population from Kasturi (drought-sensitive) × Chao Khaw (drought-tolerant). Thirteen QTLs (*qDTYs*) were identified based on two years of field data. Comparative analysis revealed two robust and consistent *DTY* QTLs, *qDTY_1.1_* and *qDTY_8.1_*, which explained the PVEs of 11.61% to 12.88% and 15.79% to 18.77%, respectively. However, *qDTY_1.1_* was found at the nearest position to the previously identified *qDTYs*. Through candidate gene analysis, the identified QTL regions in chromosome 1 (*qDTY_1.1_*) and chromosome 8 (*qDTY_8.1_*) revealed seven and five candidate genes, respectively, based on gene ontology that were significantly associated with rice grain yield-related drought traits. In conclusion, this study identified key consistent drought yield QTLs in a drought-tolerant exotic landrace. The identified QTLs provide valuable insights and resources for ongoing efforts to develop drought-tolerant rice varieties. They can be further utilized in drought breeding programs to enhance the drought resilience of existing varieties or to develop new varieties.

## Introduction

Water and food security are the most alarming concerns because of rapid climate change, and both are vulnerable to climate variability. Reports indicate that by the 21st century’s end, irregular, erratic rainfall patterns and an average global temperature of 1.4°C–5.8°C will have resulted in limited surface water availability for irrigation in field crops, resulting in drought and a reduction in agricultural yield (Misra, 2014; Sandhu et al., 2018). Furthermore, storm surges may influence freshwater availability, altering the quality of the surface irrigation water by making it saline or alkaline. Rice, a staple in millions of households, is a multiple daily meal in Asia and South America. As a cost-effective, high-calorie grain, the rice crop is a major contributor to fulfilling daily diets globally. Reports estimate that the production of rice crops will double in the next decade to satisfy the needs of an ever-growing rice-consuming population, and this must be done with less land and without depleting natural resources such as water (Sandhu and Kumar, 2017). Existing high-yielding cultivars of rice are sensitive to drought during the reproductive phase, significantly affecting grain yield. Breeding efforts to develop such varieties began more than two decades ago, utilizing both conventional and molecular approaches to integrate drought-tolerant traits into new and existing rice varieties (Bernier et al., 2007). Several studies have attempted to develop tolerant lines that do not yield under drought stress (Kumar et al., 2014; Vikram et al., 2015; Baisakh et al., 2020).

Combining the high-yield traits of irrigated rice with drought-tolerant traits from upland rice cultivators offers the potential to develop better abiotic tolerant rice varieties. Secondary drought tolerance, relative water content, canopy temperature, leaf area, and root growth were not decisive in determining grain yield under water-stress conditions. On the other hand, the grain yield-contributing traits under drought at the reproductive stage demonstrated their significance as the most reliable and efficient for selecting drought-tolerant rice genotypes (Swain et al., 2014; Vikram et al., 2015). Therefore, identification and introgression of drought QTLs is a practical approach for breeding high-yielding drought-tolerant rice (Collins et al., 2008; Kumar et al., 2008). The first reported drought QTL on chromosome 12 (*qDTY_12.1_*) was used in drought-tolerant upland and lowland rice with increased grain yield (Bernier et al., 2009; Mishra et al., 2013) because of its consistent tolerance over multiple generations (Bernier et al., 2007). Similarly, *qDTY_1.1_*, identified on chromosome 1 in the Nagina 22 variety under drought conditions, contributes to grain yield (Vikram et al., 2011; Ghimire et al., 2012; Bhattarai and Subudhi, 2018). Drought QTL *qDTY_2.3_* (Yadaw et al., 2013) from the background variety Vandana interacted with the major QTL *qDTY_12.1_*.

The effects of the identified QTLs are not universal; only a few robust QTLs work in several backgrounds, but not all. Due to epistatic interactions, the same favorable QTLs in one background may not be favorable to another genetic background. The QTL’s instability in effects across different populations QTL × genetic background interaction (Q × G) and across environments QTL × environmental interaction (Q × E) has always been the bottleneck for the successful deployment of identified QTLs in molecular breeding (Courtois et al., 2003; Lafitte et al., 2004; Bernier et al., 2008; Collins et al., 2008; Yadav et al., 2019). Although many QTL reports are available on drought tolerance, novel QTLs from exotic landraces in different genetic backgrounds linked to grain yield during the reproductive stage of drought stress are limited. Therefore, an attempt was made to develop the mapping population of an exotic drought-tolerant (Chao Khaw) landrace from Loas and a drought-sensitive variety (Kasturi) to identify any novel or previously reported drought QTLs.

## Materials and methods

### Plant materials

The present study included two parents, Kasturi (Basmati 370/CRR 88-17-1-5) and Chao Khaw, and 156 families in the F_2:3_ and F_2:4_ generations. Kasturi is a semi-dwarf variety (95 cm) with a duration of 135 to 140, suited for irrigated low ecosystems, while Chao Khaw, a landrace, is identified as a promising drought tolerant variety with semi-tall stature (110 cm) and blast resistance with bold grain type, was collected from IRRI.

### Mapping population development

This study used a mapping population developed during the 2014 dry season. The mapping population was a product of a cross between Kasturi and Chao Khaw, which produced 34 F_1_ seeds. After the hybridity test, the true plants advanced during the wet season in 2014. Two hundred F_2_ individual plants were raised and forwarded to obtain F_2:3_ and F_2:4_ mapping populations. A total of 156 families of the mapping population (Kasturi × Chao Khaw) were grown in the IRRI-South Asia Hub research fields, Hyderabad, in the wet season of 2015 (2015WS) and the wet season of 2016 (2016WS) under drought stress.

### Phenotyping

Field trials for generations F_2:3_ and F_2:4_ were conducted under drought and non-stress conditions during the reproductive stage. The clay soil field was selected under optimal conditions such as proper drainage and a good percolation rate.

The seeds were sown on nursery beds; the 21 days old seedlings were transplanted into the main field under non-stress control (NS) and drought stress (DS).

The experiment (population was evaluated) was conducted in an augmented design with four checks in four blocks following all the standard packages of practices for crop management, as suggested by Vikram et al. (2011) in the field. This study adopted a drought screening protocol at the reproductive stage, as previously described by Kumar et al. (2014). Specifically, the water was drained from the field at 30 DATS–35 DATS (days after transplanting) during 2015WS and 2016WS to maintain the cyclic soil deficit until the harvest stage. The depth of the water table was measured using a PVC pipe of 110 cm in length installed randomly in the field. When the water level in the U-PVC tube was below 1 m from the soil surface, the sensitive check showed leaf rolling and tip drying. Based on this observation, life-saving irrigation was provided through flash flooding, followed by water drainage immediately after 24 h to initiate the next stress cycle.

### Data collection and statistical analysis

The study utilized phenotypic data (IRRI SES, 2015) such as days to 50% flowering (DTF), plant height (PHT), no of the productive tiller (NPT), panicle length (PL), total spikelets per panicle (TGP), the total number of filled grains per panicle (TFGP), spikelet fertility percentage (SF%), plot yield (PY), biomass (BM), harvest index (HI%) from previous published data of Venkateshwarlu et al., 2022. To analyze the phenotypic performance of drought stress, the REML method was used, and the entries/lines with random effects were treated as fixed effects. BLUPs were estimated for each line with their standard error to assess the significance of the variance component due to the genotype (σ^2^g) for each trait. Further phenotypic correlations were calculated to determine trait associations in GenStat17. Based on the phenotypic expression, the grain yield reduction in the experiments was classified as mild (<30%), moderate (31%–64%), and severe stress (65%).

For molecular analysis, leaf samples from individual families in the mapping population were collected along with the parental lines. Genomic DNA was isolated using IRRI protocol (TPS buffer). 89 polymorphic SSR markers showed a significant difference between the drought-sensitive and drought-tolerant genotypes. These markers were used to screen the mapping population to generate genotypic data and construct a linkage map.

### Construction of the linkage/genetic map

JoinMap version 4.1 software was used to construct the linkage maps. A mapping algorithm, with a recombination frequency of 0.4 at an LOD value of 2.5 was used to group the polymorphic markers into their respective linkage groups. The Kosambi map function was used to estimate the map distance from the recombination fraction and construct a genetic map. Upon constructing genetic maps with ordered markers, the unplaced markers were seamlessly incorporated into distinct linkage groups at recombination frequencies of up to 50% using the ripple command.

### Genomic region identification and their visualization

QTL Cartographer version 2.5 was used to precisely identify the location of each QTL based on its LOD peak and the adjacent region. Mapchart 2.30 was used to visualize the identified QTL positions and linkage groups.

### Mining of candidate genes within the QTLs

Candidate genes within the identified QTL region were retrieved using the IRRI galaxy resource (http://galaxy.irri.org/). Following this, they were functionally characterized into various categories using WEGO (Ye et al., 2006) and visualized in MapMan 3.6.0 RC1 (Thimm et al., 2004). Furthermore, literature mining was performed to identify potential candidate genes related to drought tolerance within the identified QTL.

## Results

### Variability observed for grain yield and yield-related traits under reproductive stage drought stress

The population derived from the Kasturi × Chao Khaw showed variation in their phenotypic responses, such as leaf rolling, drying, and wilting symptoms, during the drought imposition at the reproductive stage of the rice crop, along with the yield and yield-contributing traits. The summary statistics and genetic parameters of the mapping population in the non-stress (NS) and drought stress (DS) conditions in 2015WS and 2016WS are presented in **Table 1**. The results revealed a drastic reduction in the grain yield of the parents and mapping population under drought stress compared to non-stress conditions in both years. It was also observed that the severity of the imposed drought during 2015WS was much higher than that during 2016WS.

**Table 1:** Summary statistics of various yield and its component traits under non-stress and drought stress conditions for Kasturi × Chao Khaw derived population.

Traits	Mean ± SEM		Min		Max		H^2^ (%)	
	2015NS	2015DS	2015NS	2015DS	2015NS	2015DS	2015NS	2015DS
DTF (days)	94 ± 0.24	90 ± 0.25	88	86	101	94	92.03	73.81
PHT (cm)	88 ± 0.57	75.44 ± 0.51	75.6	66.86	108.19	81.97	98.25	75.2
NPT	7 ± 0.1	6 ± 0.06	5	5	9	7	81.02	79.95
PL (cm)	21 ± 0.11	20 ± 0.1	19.52	18.95	23.57	23.16	68.23	73.14
PY(g)	263 ± 3.59	104 ± 3.01	201.9	55.8	344.3	143	83.03	82.72
BM (g)	587 ± 11.82	270 ± 7.91	370	120.3	1154.2	497.2	90.68	85.16
HI (%)	45 ± 0.52	39 ± 0.85	28.3	28.37	51.1	52.58	78.02	80.91
TSP	155 ± 1.93	120 ± 1.74	104	86	220	154	90.48	77.85
TFGP	138 ± 1.81	63 ± 0.92	103	42	186	85	82.07	85.35
SF%	88 ± 0.43	52 ± 0.34	75.54	48.73	95.19	57.1	81.69	84.93
GY (kg/ha)	3,291 ± 44.9	1,308 ± 37.7	2524	698	4304	1788	83.03	82.72
	2016NS	2016DS	2016NS	2016DS	2016NS	2016DS	2016NS	2016DS
DTF (days)	99 ± 0.33	89 ± 0.53	91	72	106	103	91.88	93.48
PHT (cm)	109 ± 0.66	86 ± 0.66	89.5	71.48	126.9	105.99	92.62	91.62
NPT	8 ± 0.12	6 ± 0.08	6	5	10	9	75.9	86.82
PL (cm)	24 ± 0.12	22 ± 0.13	23.05	19.91	26.11	24.14	61.41	72.43
PY (g)	362 ± 4.25	112 ± 2.34	206.6	69.2	495.7	191.8	83.55	90.64
BM (g)	666 ± 10.76	299 ± 9.54	476.3	161.7	1172.8	589.2	81.25	89.07
HI (%)	55 ± 0.85	41 ± 1.09	34.17	18.69	64.43	58.27	82.49	89.28
TSP	165 ± 2.29	126 ± 1.35	127	105	246	168	88.56	85.15
TFGP	142 ± 2.05	73 ± 0.89	102	54	209	93	83.72	85.36
SF%	86 ± 0.54	57 ± 0.4	70.06	48.47	96.45	63.29	62.25	82.81
GY (kg/ha)	4,017± 53.1	1,411 ± 29.3	2154	865	4950	2397	83.55	90.63

DTF, Days to flowering; PHT, Plant height; NPT, Number of productive tillers; PL, Panicle length; PY, Plot yield; BM, Biomass; HI, Harvest index; TSP, Total number of spikelets per panicle; SF%, Spikelet fertility percentage; TFGP, Total number of filled grains per panicle; GY, Yield in kg/ha, Mean ± SEM, Standard Error of Mean, H^2^ (%), Heritability in %.

**Figure 1:**
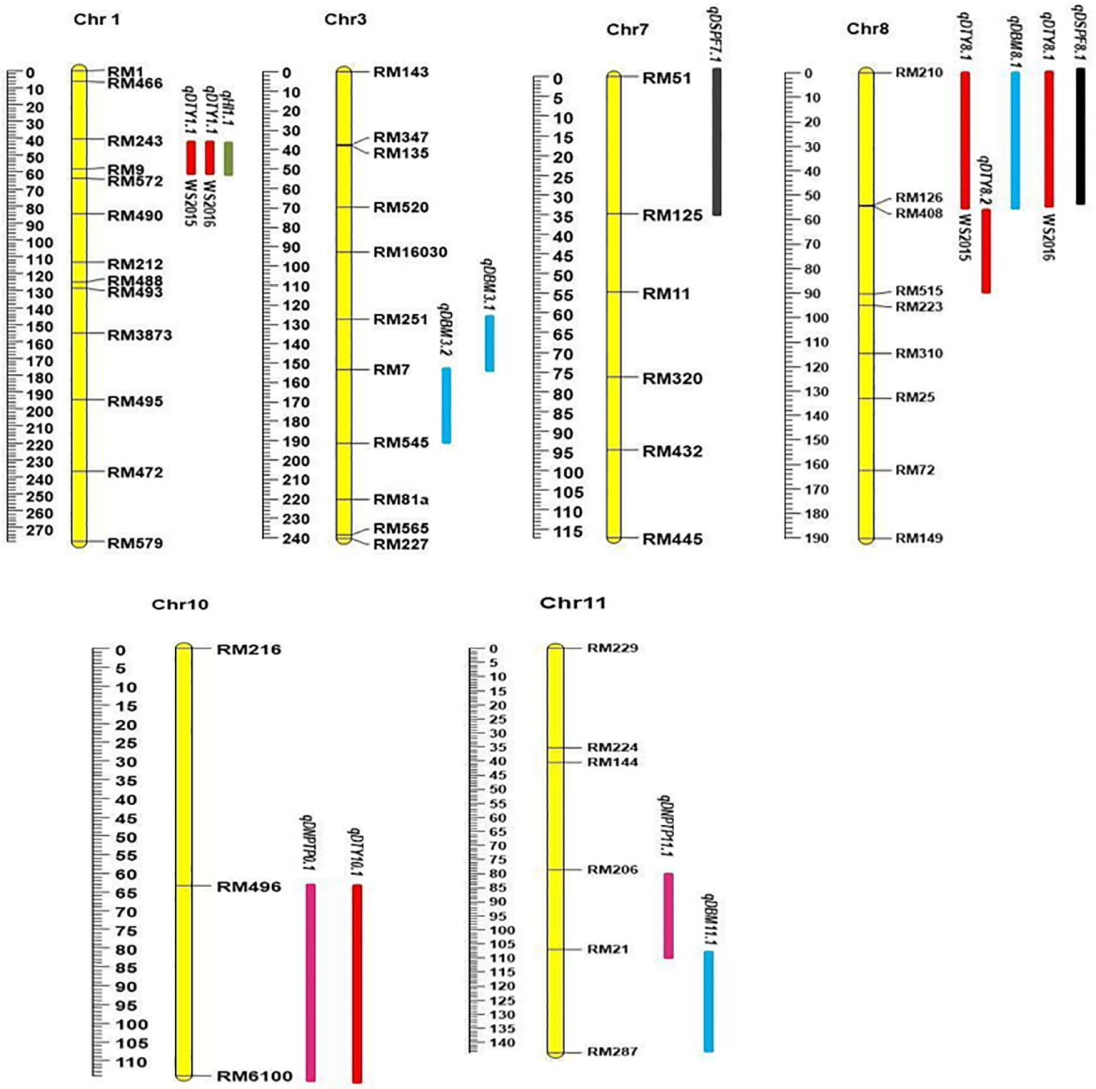
Histogram of different traits of mapping population with parents under drought stress during 2015WS and 2016WS.

The mapping population range for grain yield (kg/ha) in the drought stress and non-stress conditions in 2015WS-NS (2,524 kg/ha–4,304 kg/ha), 2015WS-DS (698 kg/ha–1,788 kg/ha), 2016WS-NS (2,154 kg/ha–4,950 kg/ha), and 2016WS-DS (865 kg/ha–2,397kg/ha), respectively. High heritability, exceeding 80%, was observed, which made the study conducive to potent QTL mapping. The population showed a normal distribution in the 2015WS and 2016WS drought stress conditions, with superior and inferior transgressive segregants (**Figure 1**).

### Genotyping of F_2:3_ population and segregation analysis

In this study, 721 microsatellite markers were utilized for the polymorphism, 89 of which showed polymorphism between the parents. The entire set of 156 F_2:3_ populations was genotyped and scored for segregation using 89 codominant SSR polymorphic markers. Polymerase Chain Reaction (PCR) product sizes of these markers ranged from 100 to 320 bp. The F_2:3_ population was scored for the allele they carried at a particular locus, i.e., the Kasturi or Chao Khaw allele. Some F_2:3_ populations amplified both alleles and were scored as heterozygous at that specific locus. In some instances where amplification failed, the data point was recorded

### QTLs controlling the yield and yield-related traits

Linkage analysis with 89 markers generated a map of 2,118.29 cM in length, where the average distance between adjacent markers was 23.80 cM. Chromosome 1 was the longest and chromosome 12 was the smallest. Chromosome 1 had the maximum number of linked markers (13), followed by chromosome 3 (11), and the minimum number of linked markers (3) was allotted to chromosome 10. The maximum map length (278.20 cM) was observed on chromosome 1, followed by chromosome 2 (248.93 cM). Chromosome 12 had the minimum map length (113.03 cM). Despite repeated polymorphism surveys with additional markers to identify the physical gaps, chromosomes 4 and 10 showed significant gaps of 40 cM and 38 cM, respectively. Gaps were found in the chromosomal region identified between the parents (**Supplementary Figure 1**).

During the 2015 and 2016 wet seasons (WS), QTLs for grain yield and related traits were identified.

**Figure 2:**
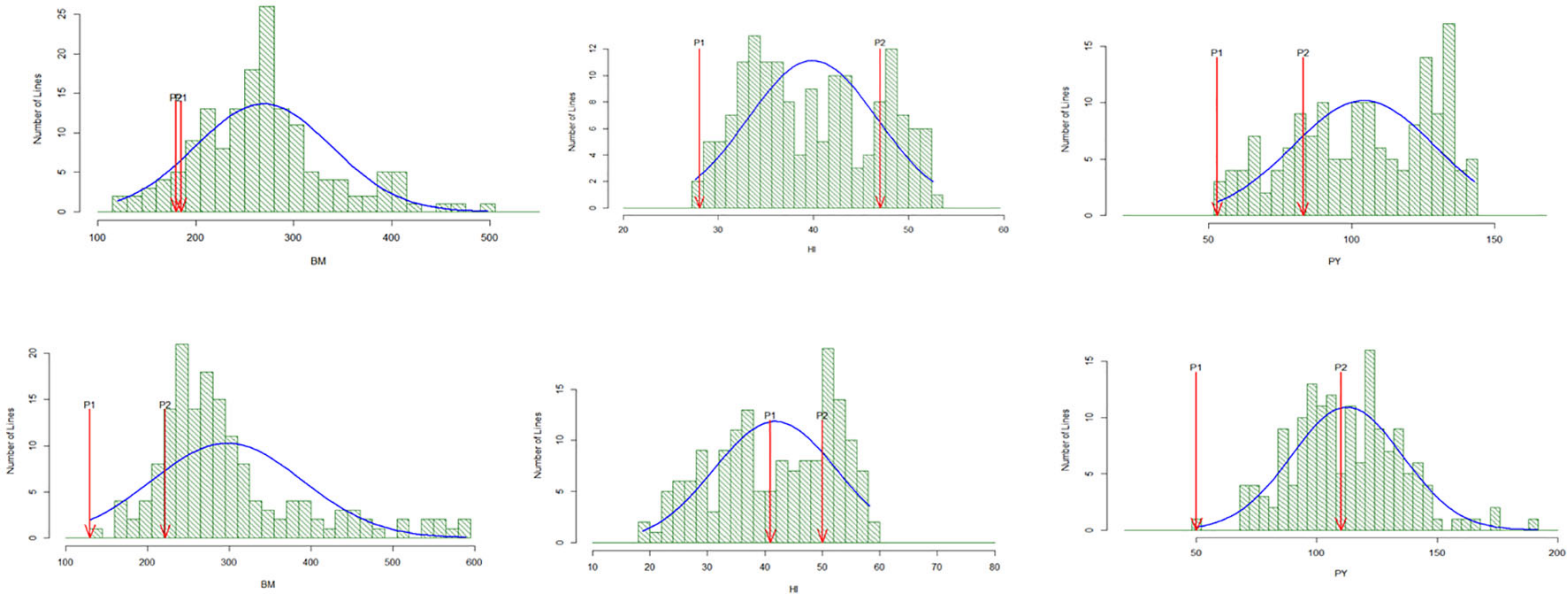
Genetic map and distribution of QTLs associated with reproductive stage drought stress in the crossbreed Kasturi × Chao Khaw. The genetic distance (centiMorgan; cM) is indicated on the left scale. Black lines within the linkage groups denote the genetic positions of markers. Six linkage groups (Chr 01, Chr 03, Chr 07, Chr 03, Chr 08, Chr 10, and Chr 11) exhibit 13 QTLs governing grain yield and yield-related traits under reproductive stage drought stress.

A total of 13 QTLs were identified during 2015WS and 2016WS together: nine QTLs for yield-attributing traits, namely, the number of tillers per plant (NPT), biomass (BM), harvest index (HI), and spikelet fertility percentage (SF%) at the reproductive stage of drought stress (**Table 2**), and four QTLs for grain yield (GY) were found with two major QTLs (*qDTY_1.1_* and *qDTY_8.1_*) with 11.61% and 18.77% phenotypic variance explained (PVE), and two minor QTLs (*qDTY_8.2_* and *qDTY_10.1_*_)_ with 6.77% and 3.28% PVE. For the number of tillers per plant (NPT), two QTLs, *qNPT_10.1_* and *qNPT_11.1_*, were identified on chromosomes 10 and 11 during the 2015 wet season with LOD scores of 3.2 and 3.2, and PVE 3.81 and 7.77%, respectively. Two QTLs, *qDBM_8.1_* and *qDBM_1.1_* for biomass (BM) identified, are located on chromosomes 8 and 11 with LOD scores of 3.5 and 2.6 and PVE of 7.97% and 8.97%, respectively. The major QTL for the harvest index (HI), *qHI_1.1_*, with a PVE of 14.67%, was identified on chromosome 1. Only one QTL for spikelet fertility percentage, *qSPF_7.1_*, with a PVE of 3.41%, was observed on chromosome 7 (**Figure 2**). QTLs were identified for grain and related traits during 2016WS, during the reproductive stage of drought stress. Six QTLs for yield and associated traits were observed and recorded during the 2016WS under stress conditions (**Table 2**). Two QTLs for DTY chromosomes 1 and 8 (*qDTY_1.1_* and *qDTY_8.1_*) were identified as major QTLs with 12.88% and 15.79% PVE and LOD values of 3.5 and 4.0. One minor QTL (*qDTY_10.1_*) was also identified on chromosome 10, with 3.28% PVE. For BM, two QTLs on chromosome 3, *qBM_3.1_* and *qBM_3.2_*, were identified with 7.42% and 6.90% PVE, and their LOD values were 9.5 and 12.0, respectively. Similarly, only one QTL, *qSPF_8.1_*, with a PVE of 5.56%, was observed (**Table 2**). Thirteen QTLs for yield and yield-related traits of drought under reproductive stress were identified. The study was conducted during 2015WS, and two QTLs were observed on chromosomes 1 and 8. *qDTY_1.1_*, between the marker interval RM243-RM9 with PVE 11.6%, is a major QTL found on chromosome 1 (**Supplementary Figures 2**, **3**).

**Table 2:** List of QTLs identified under drought stress environments during 2015WS from the mapping population Kasturi × Chao Khaw.

Season	Trait	QTL	Chromosome	Left marker	Right marker	Marker Interval (cM)	LOD	PVE (%)	Additive effect
2015WS	GY	*qDTY_1.1_*	1	RM243	RM9	17.1	4.3	11.61	9.85
2016WS	GY	*qDTY_1.1_*	1	RM243	RM9	17.1	3.5	12.88	9.97
2016WS	GY	*qDTY_10.1_*	10	RM496	RM6100	50.6	3.2	3.28	−4.72
2015WS	GY	*qDTY_8.1_*	8	RM210	RM126	54.2	7.8	18.77	13.04
2016WS	GY	*qDTY_8.1_*	8	RM210	RM126	54.2	4	15.79	11.08
2015WS	GY	*qDTY_8.2_*	8	RM408	RM515	35.7	3.2	7.71	7.6
2015WS	NPT	*qDNPT_10.1_*	10	RM496	RM6100	50.6	3.2	3.81	−0.15
2015WS	NPT	*qDNPT_11.1_*	11	RM206	RM21	28.3	3.2	6.77	0.21
2015WS	SPF%	*qDSPF_7.1_*	7	RM51	RM125	34.9	3.5	3.41	0.45
2016WS	SPF%	*qDSPF_8.1_*	8	RM210	RM126	54.2	2.5	5.56	0.78
2015WS	BM	*qDBM_11.1_*	11	RM21	RM287	36.5	2.6	8.97	24.49
2016WS	BM	*qDBM_3.1_*	3	RM251	RM7	25.9	9.5	7.42	28.94
2016WS	BM	*qDBM_3.2_*	3	RM7	RM545	37.8	12	6.9	25.45
2015WS	BM	*qDBM_8_*.*_1_*	8	RM210	RM126	54.2	3.5	7.97	23.62
2015WS	HI	*qDHI_1.1_*	1	RM243	RM9	17.1	4.2	14.67	3.12

NPT, Number of productive tillers; GY, Grain yield (g); BM, Biomass (g); HI, Harvest index (%); SF%, Spikelet fertility percentage; PVE (%), phenotypic variability explained.

### Genes underlying QTL regions

Candidate gene-based analysis resulted in genes that can be used as drought tolerance genes in future drought tolerance breeding programs. The genes were categorized based on gene ontology to filter down the genes in QTL regions that were significantly associated with the trait. The GO IDs used to filter the genes were GO:0009628 (response to abiotic stimulus), GO:0009650 (stress response), and GO:0009607 (response to biotic stimulus), as these are the primary functional categories of a gene acting in a drought environment. The genomic regions in the two major QTLs identified in chromosomes 1 and 8 were filtered down to seven and five genes, respectively. From an initial pool of 257 genes on chromosome 1, eight genes have been identified for their specific responsiveness to stress-related functional categories.

Within the QTL region on chromosome 8, comprising 1,176 genes, a focused filtration process, that is, Gene Ontology (GO) annotations, was classified into three main categories: biological processes (BP), which describe the biological objectives or processes the gene products contribute to; molecular functions (MF), which detail the specific biochemical activities or tasks performed by the gene products; and cellular components (CC), which identify the locations within the cell where the gene products are active, and has identified 47 genes associated explicitly with abiotic stress within their functional categories. Furthermore, in Kegg Pathway analysis, Ashburner et al. (2000) showed an interesting addition of genes involved in amino sugar and nucleotide sugar metabolism and phenylpropanoid biosynthesis, which abruptly reported a primary metabolic pathway involved in abiotic stress (**Table 3**).

**Table 3:** List of major QTLs and candidate genes identified under drought stress conditions during 2015WS and 2016WS.

Trait	QTLs	Chromosome	Left marker	Right marker	PVE range	Candidate gene
Grain Yield	*qDTY_1.1_*	1	RM243	RM9	11.61–12.88	*Os01g41450.1*
						*Os01g41510.1*
						*Os01g41550.1*
						*Os01g41834.1*
						*Os01g41900.1*
						*Os01g42960.1*
						*Os01g43150.1*
						*Os01g43180.1*
Grain Yield	*qDTY_8.1_*	8	RM210	RM126	15.79–18.77	*Os08g0518800*
						*Os08g0518900*
						*Os08g0519300*
						*Os08g0487800*
						*Os08g0500700*

## Discussion

Rice is a highly susceptible to drought stress at the reproductive stage, which leads to yield losses compared to other cereals, such as wheat, maize, sorghum, and pearl millet. Unlike traditional cultivars cultivated in specific rice ecosystems before the Green Revolution, modern high-yielding varieties have replaced adaptable varieties. However, the newly introduced varieties lack drought tolerance (Bernier et al., 2008; Kumar et al., 2014). Over the past two decades, the impact of climate change has intensified and become visible. Research on drought tolerance breeding has accelerated, leading to the discovery of many drought QTLs/genes.

For successful drought-tolerant breeding programs and subsequent drought-tolerant varieties, breeders need to identify droughts with significant and consistent effects across diverse environments and genetic backgrounds. Identifying QTLs/genes is crucial for integrating these findings into breeding programs. The International Rice Research Institute led this initiative. Over the past decade, IRRI has discovered several grain yield-related QTLs, such as *qDTY_1.1_*, *qDTY_2.1_*, *qDTY_3.1_*, and *qDTY_12.1_*, utilizing SSR markers (Solis et al., 2018). Farmers from Southeast Asian countries, particularly in India, Nepal, Malaysia, and Lao PDR, widely cultivate improved lines developed using mega varieties such as Swarna, Swarna-Sub1, IR64, Samba Mahsuri, Vandana, Anjali, Kalinga III, MTU1010, Savitri, MR219, and TDK1-Sub1 (Kumar et al., 2020). However, drought yield QTL is a quantitative and complex trait. Phenotypic variation explained (PVE) by the identified QTL is often limited, generally below 40%. In addition, many drought-yield QTLs show limited or no expression in other varietal backgrounds and environmental conditions. Therefore, breeders must explore novel and previously reported QTLs from new genetic backgrounds and donors to enhance their widespread application in global and national breeding programs.

This study was designed to identify QTLs/genes for yield under reproductive drought stress in a population derived from an exotic landrace from Laos and an aromatic variety, Kasturi. We provided three stress cycles during the crop duration to the two seasons of the drought trials to expose the mapping population to the maximum drought conditions. In addition, we sowed the drought trials late to adjust in such a way as to avoid the rainfall period, distinguishing between drought-tolerant and drought-sensitive lines. Bernier et al. (2007) classified lowland drought screening experiment results based on yield reductions as mild (30%), moderate (31%–65%), and severe drought (65%–85%). Kasturi experienced nearly 80% reduction in both stress trials in the drought-sensitive parent. This reduction in Kasturi yield validates the severity of the two stress trials. With an average population yield reduction exceeding 60%, these conditions were ideal for selecting drought-tolerant lines and differentially expressing lines for QTL mapping in drought-tolerant breeding. The observed high heritability, surpassing 80% for all traits at the reproductive stage under drought stress conditions, indicates a vast genetic influence on these traits. The high heritability enhances the reliability and significance of QTL mapping efforts, providing precise insights into the genetic factors that determine the yield and yield-attributing traits of drought stress. The effectiveness of yield drought QTLs is further exemplified by the successful incorporating of these favorable *qDTY* alleles into high-yielding rice varieties.

In the context of marker-assisted breeding (MAB), genetic mapping of the reproductive stage will facilitate the breeding of cultivars suitable for drought-prone environments. While numerous drought QTLs, various yield QTLs under drought stress, and various yield QTLs under reproductive-stage drought stress have been identified for the marker-assisted breeding program, the study identified a gap in QTLs from an exotic landrace controlling different traits related to drought stress tolerance. The mapping of QTLs linked to reproductive stage grain yield traits in the evaluated F_2:3_ mapping population, characterized by wide variations in ten morpho-physiological traits during stress, addresses this gap.

Genotyping of the F_2:3_ population using 721 SSR primers revealed 89 polymorphic markers, contributing to the construction of a comprehensive linkage map covering 2,118.29 cM with an average distance of 23.80 cM. In our study, several key traits associated with grain yield were identified across various chromosomes, providing better insight into enhancing the productivity of rice under drought conditions. A total of 13 QTL loci related to drought tolerance traits distributed on chromosomes 1, 3, 7, 8, 10, and 11 may provide important genetic resources for future breeding. A consistent major QTL for yield, *qDTY_1.1_*, was identified on the chromosome in 2016WS and 2016WS. Earlier studies by Ghimire et al. (2012) and Vikram et al. (2011) reported *qDTY_1.1_* on chromosome 1. Another major QTL, *qDTY_8.1_*, was identified on chromosome 8, which aligns with the results reported by Prakash et al. (2019). The study revealed that the QTLs for drought-related traits in rice overlap on chromosome 8 and positively influence the grain yield and harvest index. It is also noteworthy that the genomic region overlapped by *qDHI_1.1_* and *qDTY_1.1_* on chromosome 1, while *qDTY_8.1_, qDSPF_8.1_*, and *qDBM_8.1_*, on chromosome 8. The study identified promising lines with the high yielding are carrying the consistent QTLs under both non -stress and drought stress conditions (**Table 4**). The genes within the identified QTL region and their influence on yield were considered candidate genes. *qDTY_1.1_* is associated with genes such as *Os01g41450.1, Os01g41510.1, Os01g41550.1, Os01g41834.1, Os01g41900.1, Os01g42960.1, Os01g43150.1*, and *Os01g43180.1*. Similarly, five candidate genes (*Os08g0518800, Os08g0518900, Os08g0519300, Os08g0487800*, and *Os08g0500700*) were identified on chromosome 8.

**Table 4:** Promising high-yielding lines carrying QTLs identified under both non-stress and drought-stress conditions over parents and drought-tolerant checks during 2015WS and 2016WS.

Sl. No.	Designation	*qDTY_1.1_*	*qDTY_8.1_*	*qDTY*_8.2_	q*DNPT_10.1_*	q*DNPT_11.1_*	*qDBM_8_*.*_1_*	*qDBM_11.1_*	*qDHI_1.1_*	Remarks
1	IR 128807-38-B			Yes		Yes				
2	IR 128807-16-B		Yes	Yes			Yes			
3	IR 128807-127-B			Yes						
4	IR 128807-99-B	Yes		Yes					Yes	
5	IR 128807-82-B			Yes						
6	IR 128807-46-B			Yes						
7	IR 128807-3-B		Yes	Yes			Yes			
8	IR 128807-96-B	Yes		Yes		Yes			Yes	
9	IR 128807-163-B		Yes			Yes	Yes			
10	IR 128807-175-B			Yes						
11	IR 128807-17-B									
12	IR 128807-39-B	Yes		Yes					Yes	
13	IR 128807-95-B		Yes				Yes			
14	IR 128807-5-B									
15	IR 128807-11-B		Yes				Yes			
16	IR 128807-155-B	Yes							Yes	
17	IR 128807-12-B						Yes			
18	IR 128807-22-B					Yes				
19	IR 128807-147-B									
20	IR 128807-73-B									
21	IR 128807-100-B			Yes	Yes					

Linking QTLs to the underlying genes in mapping studies is crucial for the effective use of QTL regions. Hence, it is necessary to investigate whether other genes of interest are present in a particular QTL region. This study reports the genetic complexity of QTLs by clustering gene expression. Advances in genomics have facilitated the critical role of candidate genes in abiotic stress tolerance in plants (Sreenivasulu et al., 2007; Vij and Tyagi, 2007; Urano et al., 2010). Many transcription factors, enzymes, and stress-responsive element-binding factors are responsible for various stresses (abiotic) across multiple plant species (Pottorff et al., 2014). Although several QTLs influence tolerance in rice under drought stress, understanding the complex molecular mechanisms and linking each QTL to its underlying genes remains challenging. Although each gene has a different expression, more than 300 unique subtracted cDNA sequences covering diverse cellular activities and functional genes have been reported. Detailed bioinformatics analyses revealed that the drought tolerance mechanisms of the three rice lines were associated with different numbers and types of deferentially expressed genes. This suggests that various genes govern tolerance via distinct biochemical mechanisms. The most differentially expressed genes under drought conditions could contribute to drought tolerance, and QTLs show high correspondence among genomic regions (Fu et al., 2007). We observed two genes in the QTL region of chromosome 8 that aligned with the phenylpropanoic biosynthesis pathway. These genes are activated in response to stress conditions such as drought, heavy metal exposure, salinity, high/low temperatures, and ultraviolet radiation. This activation leads to the accumulation of diverse phenolic compounds with an inherent capability to scavenge harmful reactive oxygen species, among other functions. Three genes were identified to play essential roles in the amino acid biosynthesis pathway, which have been suggested by many researchers for drought conditions. On chromosome 1, all seven genes identified under functional ontologies were characterized as genes that can respond to abiotic stress and biotic stimuli.

## Conclusion

In conclusion, this study identified key stable drought yield QTLs utilizing a drought-tolerant exotic landrace. The study also identified QTLs linked to the genomic regions that can serve as a valuable genetic resource for developing drought-tolerant rice varieties. These QTLs can be integrated into the present drought breeding program to develop improved varieties.

## Data availability statement

The original contributions presented in the study are included in the article/**Supplementary Material**. Further inquiries can be directed to the corresponding authors.

## Ethics statement

(i) The plant materials used in this study were sourced from the International Rice Research Institute (IRRI) gene bank, an independent nonprofit research and educational institute. The IRRI holds the authority to collect and preserve rice germplasm globally for research purposes, and the materials used in this study have been documented and acquired through appropriate channels. (ii) Our experimental research and field studies on cultivated or wild plants strictly adhered to pertinent institutional, national, and international guidelines and legislation. It is ensured that all activities related to the collection of plant materials comply with the regulations. Specifically, the IRRI Rice GenBank operates according to the provisions set forth by the International Treaty on Plant Genetic Resources for Food and Agriculture (ITPGRFA), thus affirming its commitment to responsible and ethical practices in genetic resource management and research.

## Supplementary material

Supplementary Figure 1 Intra-specific genetic map of mapping population (Kasturi × Chao Khaw) with 89 SSR Markers covering 2118.29 cM.

Supplementary Figure 2 Composite interval mapping, showing QTL for drought grain yield RM243-RM9 & RM210-RM126 on chromosome 1 & Chromosome 8 and the LOD score (4.3 & 7.8) is on the Y-axis and relative location of marker name and distribution in cM on the X-axis and below is R2 value (11.61 & 18.77%) in 2015WS.

Supplementary Figure 3 Composite interval mapping, showing QTL for drought grain yield RM243-RM9 & RM210-RM126 on chromosome 1 & Chromosome 8 and the LOD score (3.5 & 4.0) is on the Y-axis and relative location of marker name and distribution in cM on the X-axis and below is R2 value (12.88%& 15.79%) in 2016WS.

## Glossary

%: Percent
BM: Biomass
BLUPs: Best linear unbiased predictors
BY: Biological yield
CG: Candidate gene
Chr: Chromosome
CIM: Composite interval mapping
cm: Centimeter
cM: Centimorgan
CSSL: Chromosome segment substitution lines
CV: Coefficient of variation
DAT: Days after transplanting
d.f.: Degree of freedom
DH: Double haploid
DNA: Deoxyribonucleic acid
dNTPs: Deoxynucleotide triphosphates
DTF: Days to 50% flowering
DS: Drought stress
DTY: Yield under drought stress
F2:3: F2 derived F3 population
G × E: Genotype by environment
GY: Yield in kg/h
GYD: Grain yield under drought
HI: Harvest index
kg/ha: Kilogram per hectare
LG: Linkage group
LOD: Logarithm of odds
MAS: Marker-assisted selection
Mb: Mega base pairs (10^6^ bp)
Min: Minutes
ml: Milliliter
MLT: Multi-location trials
mm: Millimeter
MS: Medium slender
MT: Million tons
NILs: Near isogenic lines
NPT: Number of productive tillers
NT: Number of tillers
NTP: New plant type
NS: Non-stress
PCR: Polymerase chain reaction
PH: Plant height
PL: Panicle length
PV: Phenotypic variance
PVE: Phenotypic variance explained
PY: Plot yield
QTL: Quantitative trait loci
REML: Restricted maximum likelihood
RILs: Recombinant inbred line
SDS: Sodium dodecyl sulfate
SP%: Spikelet fertility percentage
SNP: Single nucleotide polymorphism
SSR: Single sequence repeats
TFGP: Total number of filled grain panicles
TSP: Total number of spikelets per panicle
