# Supplementary figures and images for: Mapping genomic regions for reproductive stage drought tolerance in rice from exotic landrace-derived population
Source: Front Plant Sci. 2025 Jan 7;15:1495241. doi: 10.3389/fpls.2024.1495241 (PMC11756517; doi:10.3389/fpls.2024.1495241)

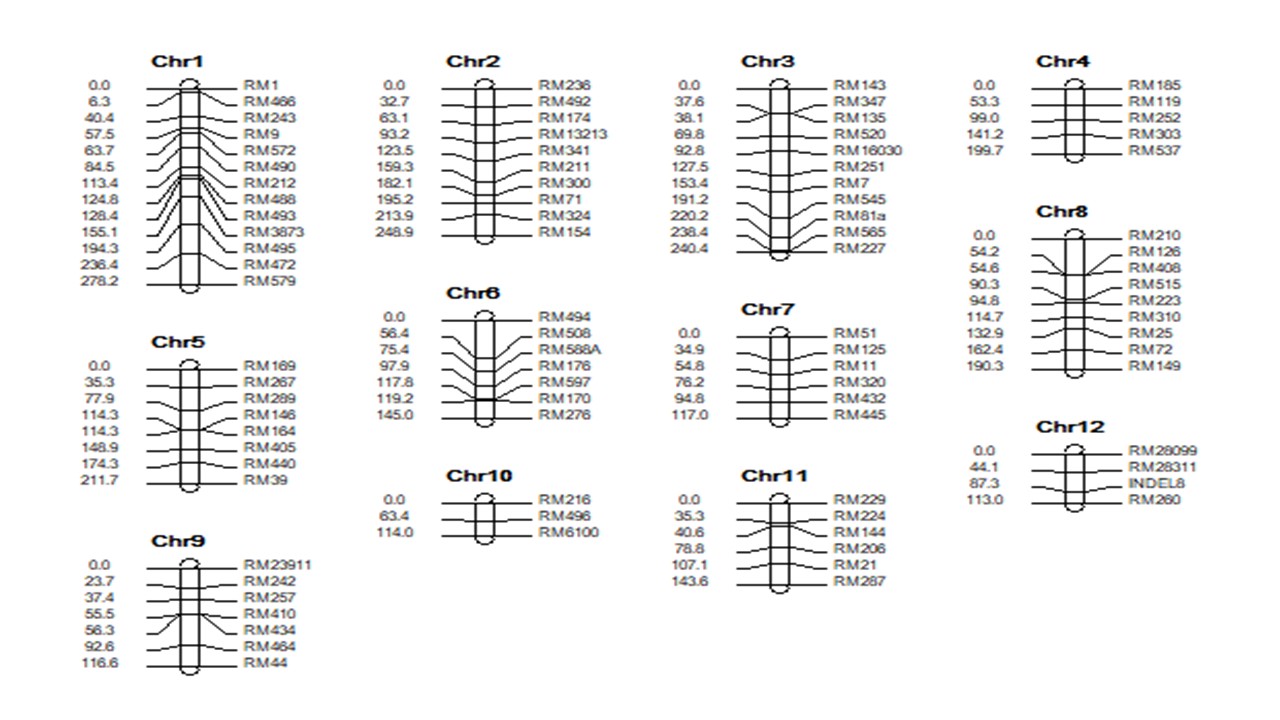

Supplement: Supplementary Figure 1 — Intra-specific genetic map of mapping population (Kasturi × Chao Khaw) with 89 SSR Markers covering 2118.29 cM. [file Image1.jpeg]

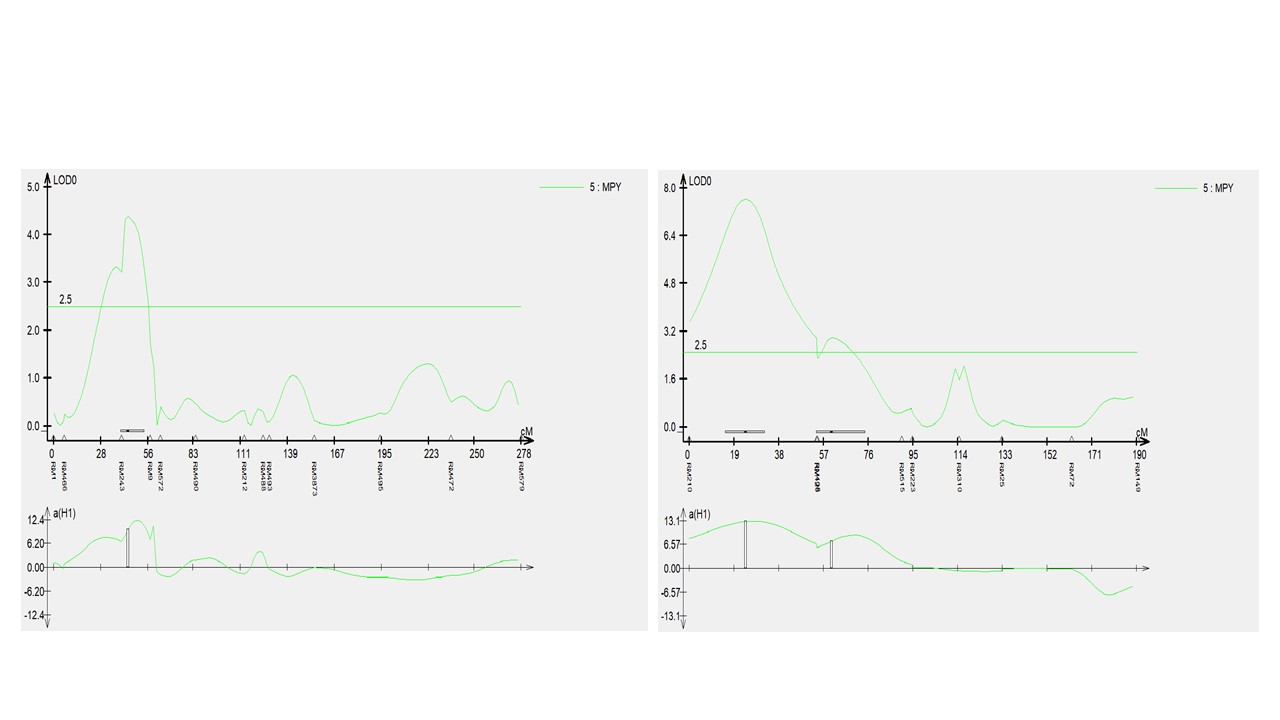

Supplement: Supplementary Figure 2 — Composite interval mapping, showing QTL for drought grain yield RM243-RM9 & RM210-RM126 on chromosome 1 & Chromosome 8 and the LOD score (4.3 & 7.8) is on the Y-axis and relative location of marker name and distribution in cM on the X-axis and below is R2 value (11.61 & 18.77%) in 2015WS. [file Image2.jpeg]

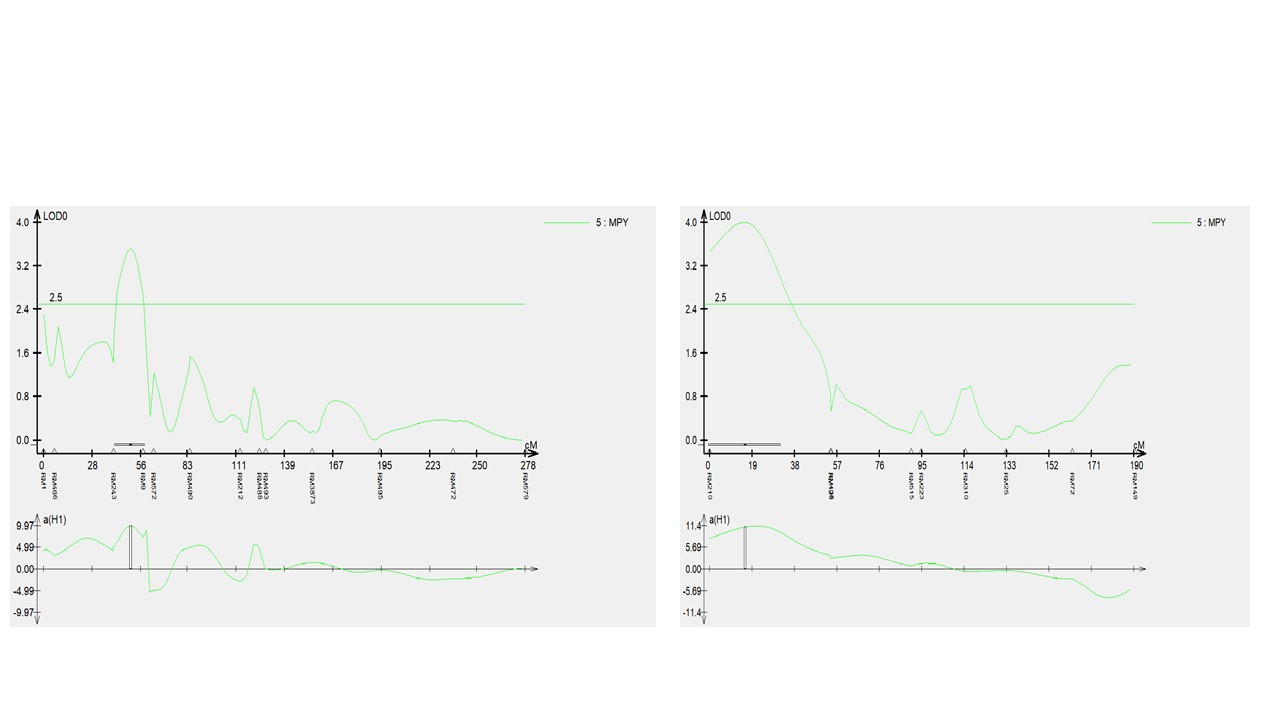

Supplement: Supplementary Figure 3 — Composite interval mapping, showing QTL for drought grain yield RM243-RM9 & RM210-RM126 on chromosome 1 & Chromosome 8 and the LOD score (3.5 & 4.0) is on the Y-axis and relative location of marker name and distribution in cM on the X-axis and below is R2 value (12.88%& 15.79%) in 2016WS. [file Image3.jpeg]
